# Leaf:wood allometry and functional traits together explain substantial growth rate variation in rainforest trees

**DOI:** 10.1093/aobpla/plz024

**Published:** 2019-04-16

**Authors:** E F Gray, I J Wright, D S Falster, A S D Eller, C E R Lehmann, M G Bradford, L A Cernusak

**Affiliations:** 1 Department of Biological Sciences, Macquarie University, New South Wales, Australia; 2 School of Biological, Earth and Environmental Sciences, University of New South Wales, Kensington, New South Wales, Australia; 3 School of Geosciences, The University of Edinburgh, Edinburgh, UK; 4 CSIRO Land and Water, Atherton, Queensland, Australia; 5 College of Science and Engineering, James Cook University, Cairns, Queensland, Australia

**Keywords:** forest ecology, functional traits, growth rate, leaf:wood allocation, plant ecological strategies, specific leaf area

## Abstract

Plant growth rates drive ecosystem productivity and are a central element of plant ecological strategies. For seedlings grown under controlled conditions, a large literature has firmly identified the functional traits that drive interspecific variation in growth rate. For adult plants, the corresponding knowledge is surprisingly poorly understood. Until recently it was widely assumed that the key trait drivers would be the same (e.g. specific leaf area, or SLA), but an increasing number of papers has demonstrated this not to be the case, or not generally so. New theory has provided a prospective basis for understanding these discrepancies. Here we quantified relationships between stem diameter growth rates and functional traits of adult woody plants for 41 species in an Australian tropical rainforest. From various cost-benefit considerations, core predictions included that: (i) photosynthetic rate would be positively related to growth rate; (ii) SLA would be unrelated to growth rate (unlike in seedlings where it is positively related to growth); (iii) wood density would be negatively related to growth rate; and (iv) leaf mass:sapwood mass ratio (LM:SM) in branches (analogous to a benefit:cost ratio) would be positively related to growth rate. All our predictions found support, particularly those for LM:SM and wood density; photosynthetic rate was more weakly related to stem diameter growth rates. Specific leaf area was convincingly correlated to growth rate, in fact negatively. Together, SLA, wood density and LM:SM accounted for 52 % of variation in growth rate among these 41 species, with each trait contributing roughly similar explanatory power. That low SLA species can achieve faster growth rates than high SLA species was an unexpected result but, as it turns out, not without precedent, and easily understood via cost-benefit theory that considers whole-plant allocation to different tissue types. Branch-scale leaf:sapwood ratio holds promise as an easily measurable variable that may help to understand growth rate variation. Using cost-benefit approaches teamed with combinations of leaf, wood and allometric variables may provide a path towards a more complete understanding of growth rates under field conditions.

## Teaser

Although plant growth rates drive ecosystem productivity and are a central element of a species ecological ‘strategy’, the chief drivers of adult growth rates under field conditions are surprisingly little understood. Here we demonstrate that substantial growth rate variation of tropical trees results from differences in relative mass allocation to leaves versus wood, and in a small number of tissue-specific properties. These results support new theory that strives to understand this topic from a cost-benefit perspective, potentially providing a way forward in a field that has largely reached an impasse.

## Introduction

Terrestrial primary productivity is a key factor controlling rates of land-atmosphere CO_2_ exchange (Beer *et al.* 2010). Tropical forests account for 34 % of global terrestrial primary productivity, a disproportionate percentage considering they cover 7–10 % of the global land surface (Lewis *et al.* 2009; Beer *et al.* 2010). Plant growth rates influence ecosystem productivity, yet the most important drivers of interspecific variation in plant growth rates remain contested (Poorter *et al.* 2008; Wright *et al.* 2010; Hérault *et al.* 2011; Paine *et al.* 2015; van der Sande *et al.* 2015).

Functional traits are morphological and physiological properties of plants that underpin variation in plant function and influence plant performance (Westoby *et al.* 2002; Adler *et al.* 2014). Two spectra of variation in leaf and wood functional traits organize species along a continuum of low to high tissue construction costs (Wright *et al.* 2004; Chave *et al.* 2009). In the case of leaves, the benefit of high specific leaf area (SLA; high leaf area deployed per unit mass construction cost) trades off against high tissue turnover rates (shorter leaf lifespans) (Reich 1998; Wright *et al.* 2004). For wood, the benefit of low tissue construction costs (low wood density) trades off against high whole-plant mortality rates (Chave *et al.* 2009; Wright *et al.* 2010). In general there is an expectation that low tissue construction costs should promote fast growth rates (Grime and Hunt 1975; Poorter and Remkes 1990; Garnier 1992; Lambers and Poorter 1992; Wright and Westoby 2001).

In seedlings this idea has found strong empirical support, particularly when considering leaf traits. Species with high SLA, high leaf nitrogen and phosphorus content, or fast photosynthetic rates, generally have faster seedling relative growth rates, at least when grown under high-resource conditions; i.e. ample light, water and nutrients (Lambers and Poorter 1992; Poorter and van der Werf 1998; Shipley 2006). However, studies examining saplings and adult plants have generally not found strong relationships between field-measured growth rates and traits, and especially not with SLA (Gower *et al.* 1993; Coomes and Grubb 1998; Poorter *et al.* 2008; Aiba and Nakashizuka 2009; Easdale and Healey 2009; Martínez-Vilalta *et al.* 2010; Wright *et al.* 2010, 2019; Hérault *et al.* 2011; Rüger *et al.* 2012; Paine *et al.* 2015). These inconsistencies have led an increasing number of researchers to conclude that those leaf traits considered to be important drivers of seedling growth rates may not be important drivers of adult growth rates (Wright *et al.* 2010, 2019; Paine *et al.* 2015). Recent studies have suggested that these inconsistencies emerge because for certain traits the strength and direction of the correlation with growth rate can change systematically as plants increase in size (Rüger *et al.* 2012; Iida *et al.* 2014; Gibert *et al.* 2016; Falster *et al.* 2018).

Leaf and wood tissue traits are unlikely to operate independently, and a means of relating these spectra is through consideration of the costs and benefits associated with the allocation of tissues to leaf or wood. In large plants, the relative amount of different tissues, perhaps even more than the tissue traits themselves, may have a decisive influence on growth rates (Ryan 1989). However, measuring total biomass allocation in large plants is difficult. An alternative is to quantify the relative costs and benefits of deploying new leaf mass versus wood mass just at the branch scale (Pickup *et al.* 2005). Those authors predicted that, all else being equal, species with relatively more leaf mass per unit wood mass sampled at the branch scale should achieve faster whole-plant growth rates. This prediction was recently confirmed in a study considering 17 woody species from a northern Australian savanna (Wright *et al.* 2019).

In this study, we consider trait–growth relationships in a very different system: a mature tropical rainforest, again in northern Australia. Focusing just on adult plants, we test predictions for how commonly studied leaf and wood tissue traits, as well as branch-scale leaf:wood ratios, should influence stem diameter growth rates of adult trees. Our expectations are outlined below, and summarized in Table 1. Traits were selected either for their comparability with the seedling growth literature, or because we had clear hypotheses for how they should drive growth rates.

**Table 1.** Predicted relationships between adult stem diameter growth rates and key leaf and wood traits, as well as branch-scale leaf:wood ratios.

Trait	Units	Definition	Expected relationship
Leaf traits			
SLA	cm^2^ g^−1^	Specific leaf area, one-sided leaf area per unit dry mass	Unrelated
*A*_area_	µmol m^−2^ s^−1^	Light-saturated photosynthetic rate, area basis	Positive
N_area_ and P_area_	g cm^−2^	Leaf nitrogen and phosphorus content, area basis	Positive
Wood traits			
Branch WD	g cm^−3^	Wood density of the sapwood in a terminal branch	Negative
Trunk WD	g cm^−3^	Wood density of the main stem	Negative
Branch leaf:wood ratios			
LM:SM	g g^−1^	Ratio of leaf mass to sapwood mass on a terminal branch	Positive
LA:SM	cm^2^ g^−1^	Ratio of leaf area to sapwood mass on a terminal branch	Positive

### Leaf tissue traits

We investigate three hypotheses related to leaf tissue traits. (i) Regardless of plant size, higher light-saturated photosynthetic rate (*A*_area_) should (all else being equal) drive faster growth rates, because faster photosynthesis increases the rate of biomass production (Gibert *et al.* 2016). (ii) We hypothesized that higher leaf N and leaf P concentrations would be associated with faster growth rates. This prediction is based on the premise that higher leaf N and P should lead to higher photosynthetic rates (Domingues *et al.* 2010) and are generally indicative of a ‘faster’ metabolic strategy (Reich 2014). (iii) Because of the large stature of our study plants, we expected that SLA and stem diameter growth rate would be unrelated, or perhaps even negatively related (Gibert *et al.* 2016). In seedlings, where leaves make up a large fraction of total biomass and leaf turnover is minimal, higher SLA should lead directly to higher growth rate because higher SLA connotes low per-area leaf construction costs. However, at increasingly larger plant sizes two effects are capable of counteracting the positive effect of high SLA and even generating an opposite trend: (i) higher SLA leaves need to be replaced more frequently (they have shorter leaf lifespans) than lower SLA leaves, and so could ultimately be more costly across a plants entire lifetime; (ii) whole-plant sapwood mass becomes a sufficiently large fraction of total biomass that the marginal cost of building sapwood to support new leaf area negates any potential growth benefits from higher SLA. That is, as plant size increases, the effect of SLA on growth rates (whether considered in terms of height, diameter or mass) diminishes and should shift from positive to unrelated, and possibly even to negative when trees are very large, or contain a very large amount of sapwood relative to leaf area (Falster *et al.* 2011, 2018; Gibert *et al.* 2016). We note that a similar prediction was made in much earlier work, based on the idea that species with longer leaf lifespans can over time generate more massive canopies than short leaf lifespan species, and thus achieve similar or even higher above-ground net productivity (Matyssek 1986; Bond 1989; Gower *et al.* 1993).

### Wood tissue traits

We hypothesized that wood density would be negatively related to stem diameter growth rates as seen in many previous studies (Enquist *et al.* 1999; Roque and Fo 2007; Poorter *et al.* 2008; Wright *et al.* 2010; Hérault *et al.* 2011; Gibert *et al.* 2016), because high wood density has a high construction cost (Hacke *et al.* 2000; Chave *et al.* 2009). Gibert *et al.* (2016) predicted that the strength of this negative correlation should be greatest in adults, because they typically have more sapwood mass (on a whole-plant basis) per unit of leaf area.

### Branch-scale leaf:wood ratios

As outlined above, we expected the relative costs of deploying new leaf area to be evident at the branch scale. All else being equal, species with relatively more leaf material on outer canopy branches were expected to have faster growth rates, and those with relatively more wood would have slower growth rates (Pickup *et al.* 2005; Wright *et al.* 2019).

In addition to testing the individual trait–growth predictions outlined above, we investigated how traits varied in relation to each other, and how traits considered in combination influenced stem diameter growth rates.

## Methods

### Growth data

Stem diameter increment data were obtained from twenty 0.5 ha permanent plots in tropical rainforest in northern Queensland, Australia, located between 145°04′E to 145°50′E and 16°08′S to 18°30′S. Plots were established between 1971 and 1980 to provide long-term ecological and growth data (Bradford *et al.* 2014). The plots range in mean annual rainfall from 1200 to 3500 mm, and in elevation from 15 to 1200 m above sea level. Besides minor selective logging on two plots before establishment, all plots are unlogged and have been protected since their establishment. The data set comprises over 10 700 individual trees, with 481 species. To estimate growth rates reliably, it is necessary to have a sufficient number of individuals measured within a species. For this reason, we focused on 41 species **[****see Supporting Information—Table S1****]** based on their abundance in the data set. Of these, 24 were chosen because they were the most abundant in the data set (diameter increments were measured on at least 100 individuals); the remaining 17 species were selected because their traits had been measured previously by Falster and Westoby (2005). These 17 species had associated diameter increment data from a minimum of 57 individuals per species, and so were also relatively abundant. The vast spatial extent covered by the measurement plots makes it likely that the species that we observed to be most abundant in the data set are representative of the most abundant species in the wider landscape.

For all species, individuals ≥10 cm diameter at breast height (dbh) were measured every 2 years for a minimum of 10 years after establishment (until 1990). After 1990, re-measurements were generally carried out every 5 years. We used all of these measurements to calculate the annual diameter growth increment of each individual using the formula GR = (dbh_final_ − dbh_init_)/(y_final_ − y_init_) where GR is annual absolute diameter growth increment, dbh_init_ and dbh_final_ are diameter at breast height of individuals at the initial and final measurement dates, respectively, and y_init_ and y_final_ are the initial and final years of measurement, respectively. Before calculating annual diameter increments we removed unreasonable measurements. We considered unreasonable measurements to be those where dbh seemingly decreased >5 % over the census period, a common practise when cleaning permanent plot growth data sets (Condit *et al.* 1993). This resulted in deletion of just 91 records from a total of 24 521.

Tropical rainforests are characterized by low understory light levels, with many individuals suppressed beneath the canopy. Because most growth–trait trade-off predictions concern growth rates when resource availability is high (Wright *et al.* 2010), rather than focusing on species-mean growth rates we instead chose to characterize species-level growth rate at a standard higher percentile of observed values. That is, we used the 95th percentile of annual diameter increments considered across all individuals of each species (hereafter referred to as GR_95_). Presumably, GR_95_ can be considered as being close to the maximum attainable growth rate for a given species (following Wright *et al.* 2010). Nonetheless, for better comparison with many previous studies, we also ran analyses using mean diameter growth rates (GR_mean_); and also the 95th percentile of diameter increments across individuals within a restricted size class (10–30 cm dbh), hereafter referred to as GR_10–30_. Note, GR here refers to absolute growth rate as is used in many studies of adult plants (King *et al.* 2006; Russo *et al.* 2010; Hérault *et al.* 2011; Poorter *et al.* 2018), whereas relative growth rate is most commonly used for studies of seedling growth (Lambers and Poorter 1992) or for adults when standardized by size (Paine *et al.* 2015).

### Trait data

#### Leaf traits

Leaf trait data for all 41 species were collected in and around Danbulla National Park in far northern Queensland (situated at ~17°07′30″S and 145°37′30″E, within the area encompassed by the permanent plots) in October 2013 and May 2014. All leaf trait measurements were made on outer canopy leaves to reduce any variation due to light environment. For three to eight adult individuals of each species [**see Supporting Information—Table S2**], we measured *A*_area_, individual leaf mass and area (for SLA), and leaf nutrient concentrations. Photosynthesis measurements were made between 08:30 am and 1:00 pm (generally before midday), on detached branches sampled from the outer canopy. Branch cut-points were immediately re-cut under water to re-establish a continuous water column, then the branch was brought to a LI-6400XT portable infrared gas analyser (LICOR Inc., Lincoln, NE, USA), fitted with 6 cm^2^ chamber with LED light source. Measurements were made under ambient CO_2_ concentrations (~400 mg L^−1^) and temperature (25–27 °C), and high light (2000 µmol m^−2^ s^−1^). Cuvette vapour pressure difference ranged between 0.61 and 1.94 kPa. Three leaves from each individual were scanned and leaf area calculated using ImageJ software (US National Institutes of Health, Bethesda, MD, USA). Leaves were oven-dried at 60–70 °C for at least 5 days and reweighed to determine dry mass. Specific leaf area was calculated by dividing leaf area by dry mass. Leaf nutrient analyses were performed at the Appleton Laboratory (University of Queensland). Leaf nitrogen concentration was determined by combustion using a LECO TruSpec CHN analyser. Leaf samples were digested in acid and total P concentration was determined by inductively coupled plasma optical emission spectrometry (ICP-OES). Leaf N_area_ and P_area_ were calculated from these data and SLA.

#### Trunk wood density

Trunk wood density (hereafter referred to as trunk WD) for all species was sourced from published (Cause *et al.* 1989; Hyland 1989) and from unpublished data (M. G. Bradford), collected previously within the study area.

#### Leaf:wood ratios and branch sapwood density

We measured leaf:wood ratios on terminal, outer canopy branches. For the 24 species sampled during the 2013–14 field campaigns, leaf:wood ratios were measured for at least five individuals from each species. Total leaf mass and wood mass were measured for stem segments at 0–5, 5–10, 10–20, 20–40, 40–80 and 80–100 cm from the tip, including biomass on any side branches extending from a segment. Fruit and flowers were generally absent, but when present they were discarded to allow direct comparison of leaf and wood material. Branch diameter was measured at each of the separation points. Leaf:wood ratios for the remaining 17 species were sampled by Falster and Westoby (2005). In that study they measured the mass of leaves and wood between the tip of the branch and the first node, and between this node and 100 cm, including all side branches. Branch diameter was measured at the node, and at 100 cm.

For samples of branch materials, we measured or calculated the following metrics: leaf mass (LM), leaf area (LA), wood mass (WM), sapwood mass (SM), leaf mass to wood mass ratio (LM:WM), leaf area to wood mass ratio (LA:WM), leaf mass to sapwood mass ratio (LM:SM) and leaf area to sapwood mass ratio (LA:SM). Total leaf area was determined by multiplying the total leaf mass by SLA. The terminal 100 cm of branches showed little evidence of leaf turnover (few leaf scars were present). Nevertheless, the leaves present at the time of measurement could potentially result from leaf accumulation minus leaf turnover. As such, we refer to these metrics as leaf:wood ratios rather than leaf:wood allocation.

There is no established standard way to express branch-scale leaf:wood ratios across a range of species, with support for sampling at a common distance from the branch tip (Falster and Westoby 2005; Wright *et al.* 2019), a common cross-sectional area (Pickup *et al.* 2005) and at the first node along a terminal branch (Westoby and Wright 2003). Because our data came from two separate field campaigns (and branches were sampled slightly differently), we were unable to use raw data at each sampling point. Instead, we estimated leaf:wood ratios at a common distance of 100 cm, as well as at a common cross-sectional area of 100 mm^2^, by interpolating between adjacent sample points. To do this, for each species the branch cross-sectional area of each individual at each separation point was plotted against leaf and wood metrics (both axes were log transformed), and leaf:wood ratios were estimated at 100 mm^2^ using the resultant regression equations. Data for several branches were discarded because their cross-sectional area was <100 mm^2^ at all sample points.

Sapwood density of branches was measured by removing a small section of branch ~10 mm in diameter and 40 mm in length, and measuring fresh volumes of the bark and sapwood by water displacement. Pith and bark were removed and branch sapwood density (hereafter referred to as branch WD) was determined by dividing dry sapwood mass by fresh sapwood volume. The relative proportions of sapwood, bark and pith were also calculated for these samples. These proportions were assumed to be approximately constant along the entirety of the branch section, allowing branch-scale sapwood mass (SM) to be estimated from total wood mass.

### Data analysis

All statistical analyses were performed in R. Any strongly right-skewed traits were log transformed; this was the case for growth rate, SLA, N_area_, P_area_ and all branch leaf:wood ratios. Normality of variables was confirmed using a Shapiro–Wilk test. For those variables that still appeared non-normal, we plotted the residuals of the linear regressions to ensure there were no major deviances from normality or homoscedasticity. Variance components analysis showed that more of the variance in SLA, N_area_, P_area_, LM:WM (at 100 cm) and branch WD was found between rather than within species. For *A*_area_, variance was split approximately equally within and between species.

Analysis of the data was a two-stage process. Firstly, we aimed to test the hypotheses laid out in Table 1 in a manner comparable to studies undertaken on seedlings. For this purpose, we used linear regressions to summarize the slope and explanatory power of individual traits for growth rates. Secondly, also of interest is which traits (singularly or in combination) can be used to capture the most variation in growth rates. To determine this, it was first necessary to understand the covariance structure in trait data. To this end, we used Pearson correlation and principal component analysis (PCA). Principal component analysis was run in the ‘prcomp’ function from the *stats* package in R. Throughout, relationships are considered significant at *P* < 0.05, but marginal significance is also noted, when 0.05 < *P* < 0.1. From our trait correlations and PCA we selected those traits best explaining the major axes of trait variation, and used forward stepwise regression using the *leaps* package (‘regsubsets’ function) to construct models to explain growth rate variation. Here we used the Bayesian information criterion (BIC) to select the most parsimonious model. The BIC estimates goodness of fit using maximum log-likelihood, and penalizes a model for increased number of parameters (Hooten and Hobbs 2015).

## Results

GR_95_ varied 9-fold between species, from 0.2 to 1.85 cm year^−1^**[****see Supporting Information—Table S1****]**. Specific leaf area varied *ca.* 5-fold among species, from 40.2 to 196.2 cm^2^ g^−1^**[****see Supporting Information—Table S2****]**. Branch WD varied the least among the measured traits (<3-fold, from 0.28 to 0.74 g cm^−3^). Branch-scale leaf and wood allocation traits showed the most variation among species, and of these total wood (including bark) mass estimated at 100 mm^2^ cross-sectional area was the most variable, ranging nearly 40-fold from 1.9 to 74.3 g **[****see Supporting Information—Tables S3** and **S4****]**.

**Figure 1.**
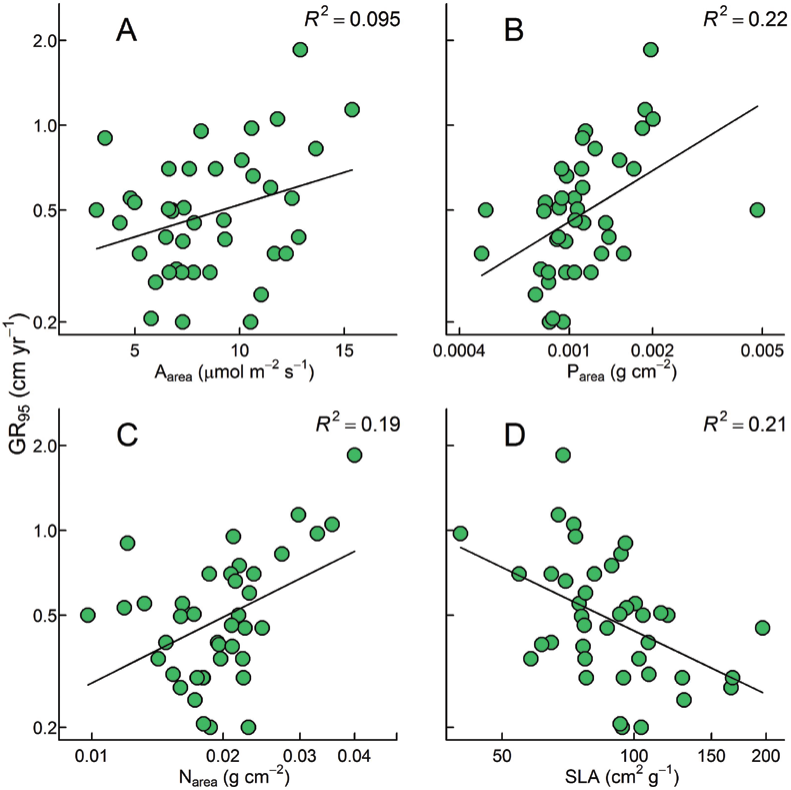
Linear regression relationships between GR_95_ and (A) *A*_area_, (B) P_area_, (C) N_area_ and (D) SLA. All variables except for *A*_area_ were log transformed. All relationships were statistically significant, *P* ≤ 0.05 **[****see Supporting Information—Table S5****]**. Relationships are for 41 tropical rainforest species (species details provided in **Supporting Information**).

All predictions regarding the direction in which traits should be related to growth rates found some support **[****see Supporting Information—Table S5****]**. As expected, *A*_area_ was positively related to GR_95_, albeit only weakly (*R*^2^ = 0.10, *P* = 0.050; Fig. 1A). Both P_area_ (*R*^2^ = 0.22, *P* = 0.002) and N_area_ (*R*^2^ = 0.19, *P* = 0.004) were more strongly and positively related to GR_95_ (Fig. 1B and C). Although removing the apparent outlier with very high P_area_ in Fig. 1B (*Acronychia acidula*) increased the *R*^2^ of that relationship from 0.22 to 0.33, we retained that data point in our analyses because we were confident that it was not erroneous (it was the mean of five similar replicate values; **see Supporting Information—Table S2**). Specific leaf area was negatively related to GR_95_, and quite convincingly so (*R*^2^ = 0.21, *P* = 0.002; Fig. 1D).

**Figure 2.**
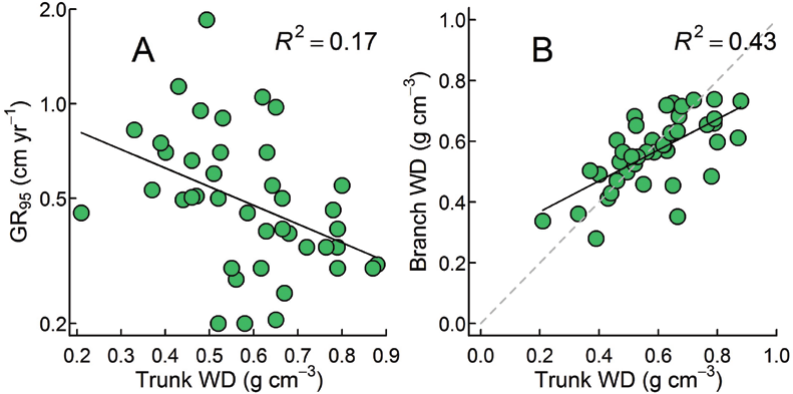
Linear regression relationships between (A) GR_95_ and trunk WD; and (B) branch and trunk WD (for comparison the 1:1 line is also shown). Only GR_95_ was log transformed. Relationships are for 41 tropical rainforest species (species details provided in **Supporting Information**).

We observed the expected negative relationship between trunk WD and GR_95_ (*R*^2^ = 0.17, *P* = 0.007; Fig. 2A). Branch WD showed a similar trend, though it was weaker and only marginally significant (*R*^2^ = 0.09, *P* = 0.054; **see Supporting Information—Table S5**). Branch WD was positively related to trunk WD (*R*^2^ = 0.43; Fig. 2B).

**Figure 3.**
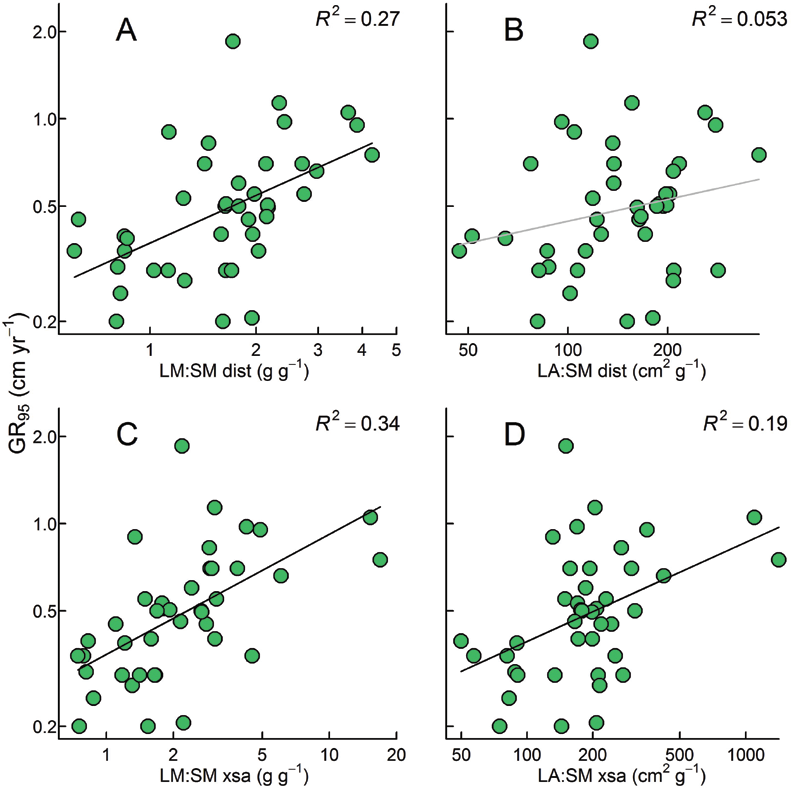
Linear regression relationships between GR_95_ and leaf:sapwood ratios expressed at a standard distance of 100 cm from the branch tip (A and B) and a standard cross-sectional area (xsa) of 100 mm^2^ (C and D). Biomass ratios are leaf mass per sapwood mass at (A) 100 mm^2^ branch cross-sectional area (LM:SM xsa); and (C) a distance of 100 cm from branch tip (LM:SM dist); and leaf area per sapwood mass at (B) 100 cm from branch tip (LA:SM dist); and 100 mm^2^ branch cross-sectional area (LA:SM xsa). All variables were log_10_ transformed. Black trend lines indicate significant regression relationships, grey lines show non-significant relationships (*P*-values reported in **Supporting Information—Table S5**). Relationships are for 41 tropical rainforest species (species details provided in **Supporting Information**).

The ratio of branch-scale leaf to sapwood mass (LM:SM, analogous to a benefit:cost ratio) explained the most variation in GR_95_ of all biomass traits **[****see Supporting Information—Table S5****]**, and was positively related to GR_95_ both at a standard distance (*R*^2^ = 0.27, *P* < 0.001; Fig. 3A) and at a standard branch cross-sectional area (*R*^2^ = 0.34, *P* < 0.0001; Fig. 3C). That is, a higher relative biomass allocation to leaf rather than sapwood mass was consistently correlated with faster growth rate.

Branch-scale leaf area:wood mass ratios explained markedly less variation in GR_95_ than did leaf mass:wood mass ratios. The difference was more pronounced when data were expressed at a common distance (Fig. 3B) than at a common cross-sectional area (Fig. 3D).

### Explanatory power of trait combinations

The second part of our analysis aimed to estimate trait covariation, and quantify growth rate variation explained by regression models with multiple traits. In doing so we aimed to identify traits which were uncorrelated, and thus captured the major axes of trait variation.

**Figure 4.**
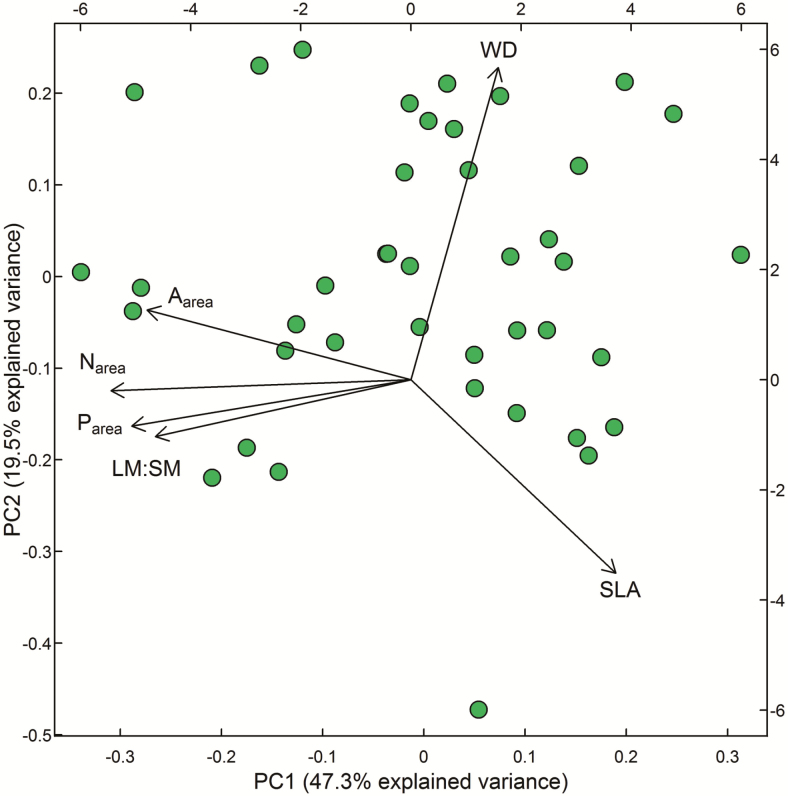
A principal component analysis showing the two main axes of variability in traits amongst 41 rainforest species. Traits are log-transformed specific leaf area (SLA), log-transformed light-saturated photosynthetic rate (*A*_area_), log-transformed leaf nitrogen (N_area_), log-transformed leaf phosphorus (P_area_), trunk wood density (WD) and log-transformed branch-scale ratio of leaf mass:sapwood mass estimated at a standard branch cross-sectional area of 100 mm^2^ (LM:SM). Each data point represents a species mean. Principal component analysis axis 1 and 2 account for 66.7 % of the variation in the data. Length of vectors represents the contribution of a trait to the ordination.

All leaf tissue traits were significantly correlated with each other (except SLA and P_area_, which were only marginally significantly correlated), while WD was not significantly correlated with other traits **[****see Supporting Information—Table S6****]**. We then explored the multivariate correlation structure among traits with a PCA fitted to species-mean data for SLA, *A*_area_, N_area_, P_area_, LM:SM (at a standard cross-sectional area) and trunk WD. The first principal axis (PC1; 47.3 % of variation; Fig. 4; **see Supporting Information—Table S7**) represented correlated variation in leaf physiology (*A*_area_, N_area_ and P_area_) and LM:SM (all negatively), and also SLA (positively, and somewhat more weakly than the other traits). The position of species along PC1 was negatively correlated to GR_95_, and more strongly than any individual trait (*R*^2^ = 0.39; **see Supporting Information—Fig. S2a**). The second principal component (19.5 % of variation; Fig. 4) represented variation in trunk WD (positively) and SLA (negatively) and was not significantly related to GR_95_**[****see Supporting Information—Fig. S2b****]**. The third axis (13.2 % variation) represented residual variation in all traits and explained 10 % of variation in GR_95_**[****see Supporting Information—Fig. S2c****]**. Considering both the axis loadings of the PCA **[****see Supporting Information—Table S7****]**, as well as the trait–trait correlations **[****see Supporting Information—Table S6****]** in combination, we observed that SLA, trunk WD and LM:SM all explained independent trait variation. On the other hand, *A*_area_, N_area_ and P_area_ were all highly correlated, and did not differentiate along any of the PC axes, except for a slight positive loading by *A*_area_ on PC2. Consequently, we retained only *A*_area_ from these three traits for the stepwise regression.

Stepwise regression of SLA, *A*_area_, trunk WD and LM:SM against GR_95_ indicated that a model including SLA, trunk WD and LM:SM was the most parsimonious (lowest Bayesian Information Criterion; Table 2). This model explained 52 % of variation in GR_95_ (*P* < 0.0001). Consistent with the bivariate results the coefficients in this model were positive for LM:SM and negative for SLA and WD. Each trait contributed approximately similar explanatory power to the model (as judged by their *t*-values having similar magnitude; Table 2).

**Table 2.** Results of the best multiple regression model to predict GR_95_, identified using forward stepwise regression. Full model included all traits (using LM:SM estimated at a standard cross-sectional area) and had a BIC of −11.9. Stepwise reduced model had a BIC of −20.5 and an *R*^2^ of 0.52, and included just SLA, WD and LM:SM.

Model terms	Coefficient	*t*	*P*
Intercept	1.15	2.39	0.02
Log (SLA)	−0.63	2.27	0.006
WD	−0.55	−2.89	0.004
Log (LM:SM)	0.22	−3.04	0.03

We used the 95th percentiles of species growth rates as our preferred growth measure, operating under the assumption that the influence of traits would be strongest in plants which were growing under more favourable conditions (Wright *et al.* 2010). As it turned out, GR_95_, GR_mean_ and GR_10–30_ were highly correlated (all *r* > 0.90, *P* < 0.0001; **see Supporting Information—Fig. S1**), and our key predictions held regardless of the growth measure used **[****see Supporting Information—Table S5****]**. In general, the strength of trait–growth relationships were slightly weaker when GR_mean_ or GR_10–30_ were used instead of GR_95_, although notably in the case of SLA the relationship was somewhat stronger (and still negative) with GR_mean_ than with GR_95_ (*r*^2^ = 0.34 vs. *r*^2^ = 0.21; **see Supporting Information—Table S5**).

## Discussion

Historically, the majority of studies on plant trait–growth relationships have focused on seedlings, as seen in existing data compilations and meta analyses (Lambers *et al.* 1990; Poorter and van der Werf 1998; Shipley 2006; Poorter *et al.* 2009; Gibert *et al.* 2016). There is now a growing literature considering saplings and adults, and a growing realization that well-established patterns in the seedling literature do not necessarily hold for plants considered at later life history stages (Gibert *et al.* 2016). Presumably, this is partly because stem diameter growth rates vary throughout ontogeny (Clark and Clark 1999; Hérault *et al.* 2011), some traits shift predictably with plant size and age (Cornelissen *et al.* 2003; Price *et al.* 2014), and the relative costs and benefits of tissue construction, turnover and physiological rates vary predictably with plant size (Ryan 1989; Gower *et al.* 1993; Gibert *et al.* 2016; Falster *et al.* 2018). This new work by Gibert, Falster and colleagues is especially promising because it outlines a theoretical framework for trait–growth relationships in relation to plant size that encompasses a variety of traits and growth measures (e.g. height or stem diameter growth, considered both in absolute and relative terms).

Based on the literature we predicted the nature of trait–growth relationships in adult plants, finding some support for all of our predictions (Table 1). Further, we showed that SLA, trunk WD and LM:SM each explained substantially independent variation in GR_95_, together accounting for 52 % of its variation. In the discussion below we focus on three particularly striking results: the negative relationship between SLA and GR_95_, the strong positive relationship between branch-scale LM:SM and GR_95_, and the combined effects of traits on GR_95_.

### SLA and its relationship to plant growth rate

Specific leaf area is a central trait in the leaf economic spectrum (Reich *et al.* 1997; Westoby *et al.* 2002; Wright *et al.* 2004); it is the conversion factor between canopy mass and canopy light-capturing area (and thus an important property in plant growth models); and it is related to important ecological variation at various scales—for example, herbivory rates, flammability and litter decomposition (Poorter *et al.* 2009). Higher SLA generally translates into faster growth in seedlings grown under non-limiting conditions (Lambers and Poorter 1992; Shipley 2006) but a growing body of literature suggests that this pattern rarely holds in adult plants (Poorter *et al.* 2008; Aiba and Nakashizuka 2009; Wright *et al.* 2010, 2019; Hérault *et al.* 2011; Iida *et al.* 2014; Gibert *et al.* 2016; Visser *et al.* 2016). Indeed, in some situations species with higher SLA may achieve *slower* growth rates. How can this be when, on face value, higher SLA should connote cheap leaf area construction and corresponding growth benefits? It seems we are only now rediscovering the mechanisms. In a literature strand from the 1970s to 1990s (Schulze *et al.* 1977; Waring and Franklin 1979; Matyssek 1986; Bond 1989; Reich *et al.* 1992; Gower *et al.* 1993), various authors stressed the central role of the leaf lifespan–SLA relationship in determining canopy development, and whole-plant (or whole-stand) productivity. That is, low SLA species with very long leaf lifespans were described as having the potential to, over time, build more massive canopies than high SLA species, with this leading to whole-plant productivity as high or even higher than that of high SLA species, despite their lower physiological rates per unit leaf mass. In that literature, the exemplar low SLA species was always an evergreen conifer, the high SLA species a deciduous angiosperm. But the principle should be the same, when considering a suite of angiosperms, all evergreen, that vary widely in SLA and leaf lifespan. Indeed, even among sclerophyllous shrubs, species with lower SLA and longer leaf lifespan may accumulate greater canopy mass per unit ground area (Read *et al.* 2006).

Gibert *et al.* (2016) and Falster *et al.* (2018) have taken this line of reasoning further, providing a mathematical formulation for understanding how SLA–growth relationships may change with plant size. The trade-off between SLA and leaf lifespan is crucial to their argument, but importantly it also considers sapwood costs per unit leaf area at a whole-plant scale, which appear to be the decisive cost that varies with plant size. Just as in the verbal models of the older literature, the Gibert/Falster theory can generate a scenario—concerning large trees—where growth rates (absolute or relative) and SLA may become negatively correlated. Indeed, this is what was found here (*r*^2^ = 0.21–0.34, depending which variant of GR was considered), in a recent study also concerning forest trees in the northern Queensland region (Wills *et al.* 2018), and in an older study of Neotropical rainforest species (Poorter *et al.* 2008). In the case of Poorter *et al.*, who considered relative growth rate (RGR) rather than absolute growth rate, the authors questioned the validity of this negative relationship. In the case of Wills *et al.*, the authors made no specific mention of the negative RGR–SLA and GR–SLA relationships reported in Table 2 of that paper. A challenge for the future is to better understand in what situations one might expect SLA–growth relationships in adult plants to be positive, negative or null.

### Leaf:wood ratios as drivers of growth rate

In general, branch biomass traits were more strongly related to stem diameter growth rates than were the various tissue traits (*A*_area_, N_area_, P_area_), with the exception of SLA. In particular, of all traits LM:SM was most strongly related to GR_95_, and this was the case for ratios expressed at a standard distance from the branch tip (100 cm, Fig. 3A) or at a given cross-sectional area (100 mm^2^; Fig. 3C). This positive relationship between branch-level leaf:wood ratios and growth rate was predicted by Pickup *et al.* (2005) and its first test—and confirmation—only recently reported (Wright *et al.* 2019). Pickup *et al.* (2005) arrived at this prediction by analogy with seedling growth equations, which most commonly decompose RGR into the product of SLA, leaf mass fraction (ratio of leaf mass to plant mass) and net assimilation rate (rate of mass increase per unit leaf area). By definition, an increase in any one of these factors must result in a proportional increase in RGR, unless the effect is counteracted by negative covariance between other terms in the equation (Wright and Westoby 2001). Pickup *et al.* (2005) argued that leaf mass fraction could also be considered at branch scale, and that species with higher branch-level leaf mass fraction would either show faster RGR at the branch scale, and/or export more photosynthate to the rest of the plant, and in either case show faster whole-plant growth rate. Our results here accord with this interpretation, and we suggest that branch-scale leaf:sapwood mass ratios could usefully be considered in future studies on trait–growth relationships. Here we calculated the various leaf:wood ratios both on a sapwood (‘SM’) basis and on a whole-stem (‘WM’) basis, and in every case the relationship with growth rate was tighter for the variant using sapwood **[****see Supporting Information—Table S5****]**. Although it takes considerably more time to remove the bark layers before measuring wood mass, our results suggest this may be time well spent.

We were uncertain about how best to express biomass ratios, and so used standardizations on both a distance and cross-sectional area basis. The area-standardized ratios in general explained more variation in GR_95_ than did the corresponding distance-standardized ratios **[****see Supporting Information—Table S5****]**. Why was this so? One possibility is that, when expressed at a standard cross-sectional area, branch-scale total leaf mass and total sapwood mass contain more independent information from one another: they are not correlated (**see Supporting Information—Fig. S3**, *R*^2^ = 0.014). By contrast, expressed at a standard distance, branch-scale total leaf mass and total sapwood mass are tightly and positively related (*R*^2^ = 0.6; **see Supporting Information—Fig. S3**); thus, each variable contains less independent information. Further investigation would be needed to verify this interpretation and, indeed, we see both methods of sampling as having their respective merits.

### Trait interactions and complementary explanatory power for growth rates

Because of the strong correlation structure among measured traits (bivariate: **Supporting Information—Table S6**; multivariate: Fig. 4), one can only go so far considering growth–trait relationships one at a time. So, what of traits in multivariate space? Here we showed that trunk WD, LM:SM and SLA each explained important, independent variation in GR_95_—and all to about the same extent (similar *t*-values), totalling 52 % explanatory power for GR_95_. Faster growth rates corresponded to higher LM:SM, lower WD and lower SLA. This is not to say that other traits were unimportant, but rather that their explanatory power for GR might have been cross-correlated with that of other traits chosen in the stepwise regressions. For example, *A*_area_, N_area_ and P_area_ were all negatively correlated with SLA, and positively correlated with LM:SM. Therefore, their effects on GR were likely tied up in both the LM:SM and SLA effects. By contrast, both the PCA and bivariate trait–trait correlations suggested that the trunk WD effect on growth rate was substantially independent from the effects of other traits. Clearly it is not straightforward to disentangle the effects of multiple cross-correlated traits in whole-plant growth outcomes, although visualization techniques such as trait correlation networks (Poorter *et al.* 2013) may be valuable.

### Trait–growth relationships: cup half full or cup half empty?

An increasing number of studies are showing that trait–growth relationships may vary systematically with plant size, and that insights from the voluminous seedling growth literature cannot be automatically applied to plants at later life history stages (Wright *et al.* 2010; Iida *et al.* 2014; Gibert *et al.* 2016; Visser *et al.* 2016; Prado-Junior *et al.* 2017). The generally low explanatory power in field-based trait–growth studies has caused particular concern (Wright *et al.* 2010; Paine *et al.* 2015). However, in both this study and one concerning savanna species (Wright *et al.* 2019), we have shown that considering traits in combination may greatly enhance the explanatory power for growth rates, to *r*^2^ values of 0.5 or higher. Is this impressive or still a cause for concern? Our view is optimistic. We are encouraged by being able to explain around half the growth rate variation in a data set, given all the ecological factors ignored—e.g. that traits and growth rates are most often measured on different individuals; that both traits and growth may vary with plant age and resource supply; that key resources such as light, water and soil nutrients may vary both in space and time; that measuring tree growth rate only in terms of trunk diameter increments ignores allometric relationships between dbh and whole-canopy mass; that below-ground biomass allocation is rarely considered.

Still it is unclear how one should best express growth rates. Our hypotheses and primary results focused on higher percentile growth rate, GR_95_, but we also reported results using mean growth rate (GR_mean_) and growth rates for plants within a restricted dbh size class (GR_10–30_). As it turned out, GR_95_ was generally better explained by traits than were the other measures, but the differences were relatively modest. Some authors choose to express tree growth rates on a relative rather than an absolute basis. The potential problem therein is that, with stem diameter appearing in the denominator, RGR is itself strongly size-dependent (Rees *et al.* 2010). One solution is to restrict sampling to trees within a constrained diameter class (Wright *et al.* 2010), or to explicitly model trait–RGR relationships as a function of tree size (Iida *et al.* 2014). Ideally, one might consider growth rates both in terms of stem diameters and height, although height growth on a relative basis presumably makes little sense. Interestingly, in the theory of Gibert/Falster *et al.*, for many of the traits they consider (including SLA, WD and *A*_area_), the same trait–growth relationships are predicted irrespective of the measure of growth: whether it is measured via increments in height or in stem diameter, or expressed on an absolute or on a relative basis.

## Conclusion

Here we found a convincing negative relationship between SLA and stem diameter growth rates, a result which is well explained by theory (Matyssek 1986; Bond 1989; Gibert *et al.* 2016; Falster *et al.* 2018), despite being opposite to that generally observed in seedlings. Leaf:sapwood mass ratios measured simply at the branch level also explained substantial variation in growth rates, suggesting that this easy-to-measure property should be included in future studies alongside traits such as SLA and photosynthetic rate. A multiple regression model including a leaf trait (SLA), a wood trait (trunk WD) and a branch biomass trait (LM:SM) was the best model for explaining variation in GR_95_. Future investigations might usefully consider how trait–growth relationships vary among habitats that differ in the maximum size of the canopy trees, or vary among species with contrasting allometric relationships between leaf and sapwood components (Ryan 1989).

## Data Accessibility

All data used in figures and tables are available in Supporting **Information**.

## Sources of Funding

E.F.G. was supported by a Macquarie University Research Scholarship and a L’Oreal-UNESCO For Women in Science Fellowship. This project was supported by Australian Research Council Discovery Funding to I.J.W., C.E.R.L. and L.A.C. (DP120103284).

## Contributions by the Authors

E.F.G. and I.J.W. conceived the ideas, designed the study and drafted the initial manuscript. E.F.G., A.S.D.E., L.A.C., D.S.F. and M.G.B. collected the data. E.F.G. analysed the data. All authors contributed critically to the drafts and gave final approval for publication.

## Supporting Information

The following additional information is available in the online version of this article—

**Table S1.** Species estimates for the three growth rate measures.

**Table S2.** Species estimates for mean tissue trait values.

**Table S3.** Species-level branch biomass metrics estimated at a standard distance of 100 cm from the branch tip.

**Table S4.** Species-level branch biomass metrics estimated at a cross-sectional area of 100 mm^2^.

**Table S5.** Details for linear regressions between traits and stem diameter growth rates.

**Table S6.** Matrix of Pearson product-moment correlation coefficients between traits.

**Table S7.** Axis loadings and explained variance of the first three components of a principal component analysis including all traits.

**Figure S1.** Linear regression relationships between GR_95_ and the two other estimates of growth rate.

**Figure S2.** Linear regression relationships between GR_95_ and principal component analysis (PCA) axes.

**Figure S3.** Linear regression relationships between leaf and sapwood components.

plz024_suppl_Supplementary_Material
