# Supplementary material for: Leaf:wood allometry and functional traits together explain substantial growth rate variation in rainforest trees
Source: AoB Plants. 2019 Apr 16;11(3):plz024. doi: 10.1093/aobpla/plz024 (PMC6510017; doi:10.1093/aobpla/plz024)
Supplement: plz024_suppl_Supplementary_Material [file plz024_suppl_supplementary_material.pdf]

Supplementary Information

Table S1 Species estimates for the three growth rate measures, namely 95th percentile of all individuals (GR_{95}), mean growth rate of all individuals (GR_{mean}), and the 95th percentile of all individuals between 10cm and 30 cm in diameter (G_{10-30}).

Species	Family	GR_{95} (n) cm yr^{-1}	GR_{mean} (± SD) cm yr^{-1}	GR_{10-30} (n) cm yr^{-1}
Alstonia muelleriana	Apocynaceae	0.25 (669)	0.09 (± 0.11)	0.25 (620)
Alstonia scholaris	Apocynaceae	0.82 (111)	0.31 (± 0.55)	0.48 (54)
Agathis robusta	Araucariaceae	0.70 (296)	0.31 (± 0.22)	0.80 (97)
Daphnandra repandula	Atherospermataceae	0.28 (474)	0.07 (± 0.12)	0.30 (408)
Gillbeea adenopetala	Cunoniaceae	0.49 (83)	0.20 (± 0.61)	0.50 (72)
Pseudoweinmannia lachnocarpa	Cunoniaceae	0.35 (582)	0.12 (± 0.61)	0.35 (502)
Pullea stutzeri	Cunoniaceae	0.50 (284)	0.17 (± 0.61)	0.50 (233)
Aleurites rockinghamensis	Euphorbiaceae	0.75 (195)	0.27 (± 0.37)	0.42 (72)
Cleistanthus myrianthus	Euphorbiaceae	0.30 (159)	0.10 (± 0.12)	0.30 (158)
Cleistanthus semiopacus	Euphorbiaceae	0.31 (1313)	0.12 (± 0.11)	0.33 (1075)
Croton insularis	Euphorbiaceae	0.35 (407)	0.14 (± 0.11)	0.35 (399)
Rockinghamia angustifolia	Euphorbiaceae	0.21 (672)	0.06 (± 0.61)	0.21 (661)
Acacia celsa	Fabaceae	1.85 (703)	0.65 (± 0.61)	1.80 (340)
Castanospora alphandii	Fabaceae	0.45 (214)	0.15 (± 0.18)	0.35 (150)
Homalium circumpinnatum	Flacourtiaceae	0.39 (294)	0.15 (± 0.61)	0.31 (248)
Cinnamomum laubatii	Lauraceae	0.51 (254)	0.16 (± 0.21)	0.32 (154)
Cryptocarya mackinnoniana	Lauraceae	0.35 (906)	0.12 (± 0.13)	0.35 (779)
Cryptocarya murrayi	Lauraceae	0.40 (189)	0.14 (± 0.15)	0.40 (168)
Endiandra leptodendron	Lauraceae	0.30 (208)	0.10 (± 0.12)	0.30 (200)
Endiandra monothyra subsp. *monothyra*	Lauraceae	0.55 (323)	0.12 (± 0.20)	0.50 (270)
Litsea leefeana	Lauraceae	0.60 (571)	0.20 (± 0.61)	0.65 (332)
Neolitsea dealbata	Lauraceae	0.40 (68)	0.15 (± 0.61)	0.41 (57)
Argyrodendron peralatum	Malvaceae	0.98 (121)	0.40 (± 0.37)	0.83 (38)
Franciscodendron laurifolium	Malvaceae	0.53 (3325)	0.19 (± 0.61)	0.45 (2257)
Steganthera laxiflora	Monimiaceae	0.30 (518)	0.07 (± 0.61)	0.25 (255)
Myristica globosa subsp. *globosa*	Myristicaceae	0.50 (975)	0.16 (± 0.61)	0.55 (774)
Gossia hillii	Myrtaceae	0.39 (365)	0.18 (± 0.61)	0.39 (365)
Syzygium sayeri	Myrtaceae	0.46 (57)	0.21 (± 0.61)	0.19 (25)
Cardwellia sublimis	Proteaceae	0.66 (613)	0.22 (± 0.21)	0.70 (372)
Darlingia darlingiana	Proteaceae	0.55 (565)	0.14 (± 0.17)	0.55 (402)
Alphitonia petriei	Rhamnaceae	1.14 (87)	0.40 (± 0.33)	1.35 (31)
Alphitonia whitei	Rhamnaceae	0.70 (666)	0.23 (± 0.22)	0.70 (466)
Acronychia acidula	Rutaceae	0.50 (310)	0.19 (± 0.18)	0.48 (282)
Acronychia laevis	Rutaceae	0.20 (934)	0.06 (± 0.09)	0.20 (934)
Brombya platynema	Rutaceae	0.20 (2087)	0.06 (± 0.08)	0.20 (2087)
Dinosperma erythrococcum	Rutaceae	0.30 (686)	0.14 (± 0.09)	0.30 (628)
Flindersia bourjotiana	Rutaceae	0.70 (1577)	0.26 (± 0.61)	0.60 (880)
Flindersia brayleyana	Rutaceae	0.95 (520)	0.32 (± 0.61)	0.59 (183)
Flindersia pimenteliana	Rutaceae	0.90 (743)	0.37 (± 0.61)	0.86 (356)

Species	Family	GR_{95} (n)<br>cm yr^{-1}	GR_{mean} (± SD)<br>cm yr^{-1}	$GR_{10\text{-}30}$ (n)<br>cm yr^{-1}
Castanospermum australe	Sapindaceae	1.05 (300)	0.31 (± 0.48)	1.06 (158)
Dendrocnide photinophylla	Urticaceae	0.45 (803)	0.10 (± 0.17)	0.37 (566)
**Mean**		**0.55**	**0.19**	**0.49**
**Minimum**		**0.20**	**0.06**	**0.2**
**Maximum**		**1.85**	**0.65**	**1.8**

Table S2 Species estimates for mean tissue trait values (with standard deviation, and number of replicates, in brackets). Mean trunk diameter of individuals sampled for traits is also indicated. Trunk wood density (Trunk WD) does not have an associated standard deviation and samples size as these values are taken from the literature.

**Species**	**Family**	A_{area} **(SD, n)** µmol m^{-2} s^{-1}	N_{area} **(SD, n)** g cm^{-2}	P_{area} **(SD, n)** g cm^{-2}	**SLA (SD, n)** cm^{2} g^{-1}	**Branch WD (SD, n)** g cm^{-3}	**Trunk WD** g cm^{-3}	**Trunk Diameter (SD, n)** cm
Alstonia muelleriana	Apocynaceae	11.04 (2.74, 7)	0.017 (0.0009, 5)	0.0008 (0.00009, 5)	129.95 (46.23, 7)	0.68 (0.047, 5)	0.67	13.0 (1.94, 6)
Alstonia scholaris	Apocynaceae	13.64 (1.28, 4)	0.027 (0.0012, 5)	0.0012 (0.00012, 5)	93.34 (3.97, 3)	0.36 (0.026, 3)	0.33	NA
Agathis robusta	Araucariaceae	6.63 (3.49, 13)	0.023 (0.0021, 5)	0.0017 (0.00030, 5)	54.69 (9.18, 7)	0.49 (0.054, 7)	0.40	30.5 (4.47, 9)
Daphnandra repandula	Atherospermataceae	5.99 (0.85, 6)	0.016 (0.0005, 5)	0.0008 (0.00013, 5)	166.35 (14.11, 6)	0.56 (0.193, 4)	0.56	16.6 (2.77, 5)
Gillbeea adenopetala	Cunoniaceae	6.77 (1.11, 5)	0.016 (0.0018, 5)	0.0008 (0.00009, 5)	75.98 (13.42, 3)	0.43 (0.049, 3)	0.44	NA
Pseudoweinmannia lachnocarpa	Cunoniaceae	5.23 (1.65, 6)	0.014 (0.0009, 5)	0.0005 (0.00014, 5)	77.35 (8.78, 6)	0.74 (0.043, 6)	0.72	16.1 (1.99, 6)
Pullea stutzeri	Cunoniaceae	3.17 (1.88, 6)	0.010 (0.0004, 5)	0.0005 (0.00008, 5)	104.76 (12.24, 6)	0.63 (0.126, 5)	0.67	15.2 (1.75, 6)
Aleurites rockinghamensis	Euphorbiaceae	10.11 (3.01, 6)	0.022 (0.0016, 5)	0.0015 (0.00016, 5)	88.93 (6.88, 3)	0.28 (0.035, 3)	0.39	10.2 (1.69, 3)
Cleistanthus myrianthus	Euphorbiaceae	7.81 (2.03, 5)	0.018 (0.0013, 5)	0.0010 (0.00010, 5)	128.88 (13.71, 3)	0.59 (0.003 3)	0.62	NA
Cleistanthus semiopacus	Euphorbiaceae	6.99 (1.29, 8)	0.015 (0.0005, 5)	0.0008 (0.00007, 5)	108.13 (6.07, 6)	0.73 (0.242, 6)	0.88	20.3 (1.95, 9)
Croton insularis	Euphorbiaceae	11.67 (1.91, 6)	0.022 (0.0008, 5)	0.0013 (0.00009, 5)	102.64 (9.19, 6)	0.66 (0.062, 6)	0.79	15.7 (0.96, 9)
Rockinghamia angustifolia	Euphorbiaceae	5.78 (3.30, 5)	0.018 (0.0007, 4)	0.0009 (0.00016, 4)	92.99 (8.82, 3)	0.45 (0.006, 3)	0.65	12.0 (2.04, 5)
Acacia celsa	Fabaceae	12.90 (1.34, 5)	0.040 (0.0032, 5)	0.0020 (0.00051, 5)	68.96 (4.13, 3)	0.50 (0.054, 3)	0.49	26.0 (5.51, 7)
Castanospermum australe	Fabaceae	11.81 (5.28, 5)	0.036 (0.0020, 5)	0.0020 (0.00026, 5)	72.88 (11.71, 3)	0.59 (0.061, 3)	0.62	NA
Homalium circumpinnatum	Flacourtiaceae	7.29 (2.78, 6)	0.021 (0.0013, 5)	0.0010 (0.00010, 5)	76.51 (15.32, 6)	0.72 (0.035, 6)	0.68	28.8 (5.85, 6)
Cinnamomum laubatii	Lauraceae	7.35 (1.75, 4)	0.016 (0.0005, 4)	0.0009 (0.00012, 4)	115.10 (6.90, 4)	0.53 (0.045, 4)	0.47	10.1 (1.43, 4)
Cryptocarya mackinnoniana	Lauraceae	12.22 (3.30, 6)	0.020 (0.0018, 5)	0.0016 (0.00033, 5)	54.87 (11.54, 4)	0.65 (0.075, 3)	0.76	19.6 (2.64, 5)
Cryptocarya murrayi	Lauraceae	12.82 (2.46, 5)	0.019 (0.0022, 4)	0.0014 (0.00024, 4)	64.74 (1.02, 3)	0.67 (0.075, 3)	0.79	21.5 (1.75, 2)
Endiandra leptodendron	Lauraceae	8.59 (3.02, 5)	0.022 (0.0009, 5)	0.0012 (0.00007, 5)	94.54 (8.87, 3)	0.61 (0.052, 3)	0.87	NA
Endiandra monothyra subsp. *monothyra*	Lauraceae	4.80 (3.10, 5)	0.016 (0.0006, 5)	0.0009 (0.00013, 5)	100.62 (12.62, 6)	0.60 (0.027, 5)	0.80	12.9 (2.29, 6)
Litsea leefeana	Lauraceae	11.48 (1.70, 60	0.023 (0.0008, 5)	0.0011 (0.00022, 5)	77.54 (7.63, 3)	0.55 (0.047, 3)	0.51	NA

Species	Family	A_{area} (SD, n) µmol m^{-2} s^{-1}	N_{area} (SD, n) g cm^{-2}	P_{area} (SD, n) g cm^{-2}	SLA (SD, n) cm^2 g^{-1}	Branch WD (SD, n) g cm^{-3}	Trunk WD g cm^{-3}	Trunk Diameter (SD, n) cm
Neolitsea dealbata	Lauraceae	6.49 (1.87, 7)	0.015 (0.0009, 5)	0.0009 (0.00022, 5)	107.75 (3.82, 3)	0.35 (0.025, 3)	0.67	4.5 (0.07, 2)
Argyrodendron peralatum	Malvaceae	10.57 (2.69, 5)	0.033 (0.0019, 5)	0.0018 (0.00037, 5)	40.22 (6.22, 3)	0.73 (0.022, 3)	0.65	9.4 (0, 1)
Franciscodendron laurifolium	Malvaceae	5.00 (1.92, 5)	0.012 (0.0013, 5)	0.0008 (0.00003, 5)	96.17 (10.84, 5)	0.50 (0.041, 5)	0.37	13.9 (1.64, 5)
Steganthera laxiflora	Monimiaceae	6.65 (1.72, 6)	0.017 (0.0006, 5)	0.0008 (0.00014, 5)	167.62 (16.43, 6)	0.46 (0.051, 6)	0.55	13.4 (1.03, 5)
Myristica globosa subsp. *globosa*	Myristicaceae	6.62 (2.90, 8)	0.017 (0.0015, 5)	0.0011 (0.00012, 5)	92.80 (11.21, 8)	0.47 (0.031, 6)	0.46	19.8 (1.78, 7)
Gossia hillii	Myrtaceae	9.31 (2.46, 6)	0.020 (0.0014, 5)	0.0009 (0.00012, 5)	61.61 (11.54, 6)	0.72 (0.010, 6)	0.63	21.5 (1.89, 7)
Syzygium sayeri	Myrtaceae	9.25 (3.12, 5)	0.021 (0.0008, 5)	0.0010 (0.00008, 5)	77.03 (7.40, 3)	0.48 (0.023, 3)	0.78	NA
Cardwellia sublimis	Proteaceae	10.65 (3.77, 5)	0.021 (0.0013, 5)	0.0010 (0.00011, 5)	69.82 (7.07, 3)	0.60 (0.046, 3)	0.46	6.0 (0, 1)
Darlingia darlingiana	Proteaceae	12.50 (3.90, 6)	0.013 (0.0014, 5)	0.0010 (0.00028, 5)	74.95 (11.79, 6)	0.63 (0.094, 6)	0.64	13.6 (1.07, 7)
Alphitonia petriei	Rhamnaceae	15.38 (3.86, 5)	0.030 (0.0021, 5)	0.0019 (0.00032, 5)	67.29 (4.29, 3)	0.41 (0.017, 3)	0.43	11.7 (2.11, 4)
Alphitonia whitei	Rhamnaceae	7.60 (2.57, 7)	0.021 (0.0017, 5)	0.0011 (0.00013, 5)	81.25 (13.51, 7)	0.57 (0.051, 6)	0.63	20.7 (2.97, 8)
Acronychia acidula	Rutaceae	6.78 (1.75, 6)	0.022 (0.0010, 5)	0.0048 (0.00130, 5)	119.31 (16.86, 3)	0.52 (0.031, 3)	0.52	16.4 (1.54, 8)
Acronychia laevis	Rutaceae	7.28 (1.46, 6)	0.019 (0.0027, 5)	0.0009 (0.00017, 5)	103.77 (28.04, 6)	0.68 (0.051, 6)	0.52	12.6 (0.58, 8)
Brombya platynema	Rutaceae	10.53 (2.38, 5)	0.023 (0.0008, 5)	0.0009 (0.00009, 5)	94.02 (2.99, 3)	0.60 (0.069, 3)	0.58	NA
Dinosperma erythrococcum	Rutaceae	7.25 (2.98, 5)	0.018 (0.0008, 5)	0.0010 (0.00007, 5)	77.82 (2.99, 4)	0.74 (0.044, 6)	0.79	20.7 (3.55, 9)
Flindersia bourjotiana	Rutaceae	8.85 (2.96, 7)	0.019 (0.0025, 5)	0.0009 (0.00014, 5)	64.78 (4.80, 6)	0.65 (0.142, 6)	0.53	26.8 (5.39, 6)
Flindersia brayleyana	Rutaceae	8.17 (4.16, 7)	0.021 (0.0025, 5)	0.0011 (0.00010, 5)	73.55 (14.35, 7)	0.56 (0.154, 6)	0.48	19.3 (3.77, 9)
Flindersia pimenteliana	Rutaceae	3.58 (1.33, 5)	0.012 (0.0014, 5)	0.0011 (0.00037, 5)	95.64 (29.47, 5)	0.55 (0.098, 5)	0.53	14.1 (2.00, 5)
Castanospora alphandii	Sapindaceae	4.29 (2.62, 6)	0.022 (0.0008, 5)	0.0014 (0.00013, 5)	86.81 (7.59, 6)	0.56 (0.055,6)	0.59	19.2 (2.51, 5)
Dendrocnide photinophylla	Urticaceae	7.84 (2.70, 7)	0.025 (0.0034, 5)	0.0011 (0.00010, 5)	196.23 (22.71, 7)	0.34 (0.006, 6)	0.21	21.3 (2.11, 8)

Table S3 Species level branch biomass metrics estimated at a standard distance of 100 cm from the branch tip (with 95 % confidence interval shown in brackets).

Species	Family	LM_{dist} [95% CI]	LA_{dist} [95% CI]	WM_{dist} [95% CI]	SM_{dist} [95% CI]
Alstonia muelleriana	Apocynaceae	21.6 [14.6; 31.9]	2663 [1983; 3576]	36.0 [26.9; 48.2]	26.2 [19.0; 36.2]
Alstonia scholaris	Apocynaceae	94.6 [45.1; 198.3]	8815 [4303; 18058]	91.0 [66.3; 124.8]	64.5 [44.5; 93.6]
Agathis robusta	Araucariaceae	130.1 [88.6; 191.1]	7029 [4680; 10558]	193.2 [110.3; 338.5]	91.1 [50.7; 163.6]
Daphnandra repandula	Atherospermataceae	33.6 [23.4; 48.1]	5567 [4010; 7729]	28.7 [20.9; 39.6]	26.8 [18.0; 39.7]
Gillbeea adenopetala	Cunoniaceae	140.8 [51.8; 382.8]	10524 [4476; 24746]	83.1 [29.0; 238.6]	65.0 [23.8; 178.1]
Pseudoweinmannia lachnocarpa	Cunoniaceae	42.3 [29.7; 60.4]	3264 [2335; 4561]	106.7 [80.1; 142.2]	69.4 [51.9; 92.7]
Pullea stutzeri	Cunoniaceae	52.8 [36.7; 76.0]	5502 [3866; 7830]	38.9 [28.8; 52.5]	29.6 [21.0; 41.9]
Aleurites rockinghamensis	Euphorbiaceae	229.1 [81.7; 642.4]	20333 [6577; 62862]	69.9 [25.9; 188.9]	53.8 [20.7; 140.1]
Cleistanthus myrianthus	Euphorbiaceae	57.3 [37.1; 88.5]	7296 [4618; 11527]	44.1 [26.5; 73.6]	34.9 [20.5; 59.5]
Cleistanthus semiopacus	Euphorbiaceae	41.5 [33.1; 52.2]	4486 [3565; 5643]	69.1 [46.3; 103.2]	51.3 [34.4; 76.5]
Croton insularis	Euphorbiaceae	31.3 [24.0; 41.0]	3207 [2440; 4214]	50.3 [38.7; 65.3]	37.0 [28.8; 47.6]
Rockinghamia angustifolia	Euphorbiaceae	68.9 [38.2; 124.3]	6398 [3167; 12928]	43.4 [26.1; 72.1]	35.4 [20.6; 60.9]
Acacia celsa	Fabaceae	76.2 [16.5; 352.9]	5200 [1063; 25431]	54.6 [14.6; 204.3]	44.3 [11.7; 167.9]
Castanospermum australe	Fabaceae	344.6 [194.5; 610.5]	24557 [13148; 45865]	130.2 [79.9; 212.3]	94.5 [57.8; 154.6]
Homalium circumpinnatum	Flacourtiaceae	55.3 [41.1 74.4]	4151 [3113; 5536]	107.0 [82.3; 139.1]	64.2 [49.6; 83.0]
Cinnamomum laubatii	Lauraceae	55.6 [39.9; 77.3]	6386 [4531; 9003]	43.4 [30.2; 62.5]	33.8 [23.6; 48.4]
Cryptocarya mackinnoniana	Lauraceae	206.0 [108.1; 392.6]	11469 [5964; 22056]	88.0 [44.1; 175.6]	101.4 [21.7; 474.4]
Cryptocarya murrayi	Lauraceae	145.4 [72.4; 292.0]	9384 [4589; 19192]	92.4 [32.6; 261.8]	74.4 [26.4; 209.6]
Endiandra leptodendron	Lauraceae	81.1 [21.9; 300.5]	7699 [1968; 30126]	88.2 [33.2; 234.5]	72.0 [27.6; 188.2]
Endiandra monothyra subsp. *monothyra*	Lauraceae	43.3 [31.8; 59.0]	4326 [3172; 5900]	32.4 [23.9; 44.1]	21.9 [15.3; 31.2]
Litsea leefeana	Lauraceae	220.5 [127.5; 381.2]	16993 [8719; 33117]	162.4 [101.5; 259.9]	123.5 [84.7; 180.1]
Neolitsea dealbata	Lauraceae	49.2 [22.3; 108.8]	5302 [2324; 12096]	41.7 [23.6; 73.9]	30.9 [17.0; 56.4]
Argyrodendron peralatum	Malvaceae	294.2 [94.9; 912.1]	11735 [3722; 37001]	210.9 [59.4; 748.5]	122.4 [33.3; 449.8]
Franciscodendron laurifolium	Malvaceae	44.9 [30.2; 66.9]	4298 [2918; 6330]	54.0 [38.7; 75.4]	36.1 [25.4; 51.4]
Steganthera laxiflora	Monimiaceae	35.7 [27.1; 46.9]	5956 [4444; 7982]	26.2 [19.1; 35.9]	20.9 [15.2; 28.9]

Species	Family	LM_{dist} [95% CI]	LA_{dist} [95% CI]	WM_{dist} [95% CI]	SM_{dist} [95% CI]
Myristica globosa subsp. *globosa*	Myristicaceae	43.1 [35.6; 52.2]	3973 [3320; 4755]	33.9 [28.1; 40.8]	20.0 [16.2; 24.6]
Gossia hillii	Myrtaceae	44.3 [32.2; 60.8]	2687 [2009; 3593]	73.4 [42.1; 128.0]	52.3 [30.3; 90.3]
Syzygium sayeri	Myrtaceae	141.1 [29.0; 686.1]	10938 [2458; 48688]	92.6 [11.1; 773.6]	65.9 [8; 538.8]
Cardwellia sublimis	Proteaceae	337.5 [251.9; 452.2]	23670 [17510; 31998]	148.5 [75.6; 291.8]	113.7 [59.4; 217.5]
Darlingia darlingiana	Proteaceae	84.9 [66.7; 107.9]	6293 [4978; 7955]	41.3 [34.5; 49.5]	31.0 [25.9; 37.2]
Alphitonia petriei	Rhamnaceae	79.7 [48.1; 132.0]	5345 [3512; 8135]	47.5 [31.2; 72.3]	34.2 [22.0; 53.3]
Alphitonia whitei	Rhamnaceae	190.7 [113.8; 319.6]	15304 [9659; 24248]	133.4 [74.7; 238.0]	70.6 [43.9; 113.5]
Acronychia acidula	Rutaceae	121.9 [43.0; 345.2]	14473 [6031; 34734]	92.6 [30.6; 280.4]	74.5 [24.5; 226.3]
Acronychia laevis	Rutaceae	35.0 [23.1; 53.1]	3535 [2235; 5592]	57.6 [37.9; 87.5]	43.6 [29.3; 65.0]
Brombya platynema	Rutaceae	38.0 [15.3; 94.4]	3571 [1432; 8908]	28.6 [12.7; 64.3]	23.6 [10.5; 53.0]
Dinosperma erythrococcum	Rutaceae	66.6 [52.2; 85.1]	5318 [3835; 7375]	85.0 [64.2; 112.5]	65.0 [49.3; 85.6]
Flindersia bourjotiana	Rutaceae	112.9 [88.2; 144.7]	7300 [5630; 9466]	75.6 [60.2; 95.1]	53.0 [42.1; 66.7]
Flindersia brayleyana	Rutaceae	86.9 [64.8; 116.6]	6284 [4606; 8573]	42.8 [31.8; 57.8]	22.5 [16.0; 31.6]
Flindersia pimenteliana	Rutaceae	38.9 [22.7; 66.7]	3598 [1897; 6826]	48.2 [31.1; 74.9]	34.4 [22.6; 52.3]
Castanospora alphandii	Sapindaceae	89.0 [65.9; 120.2]	7683 [5657; 10433]	71.6 [42.1; 121.8]	46.8 [27.5; 79.7]
Dendrocnide photinophylla	Urticaceae	16.8 [11.6; 24.2]	3270 [2269; 4714]	36.0 [26.9; 48.4]	26.7 [18.9; 37.8]

Table S4 Species level branch biomass metrics estimated at a cross sectional area of 100 mm^2 (with 95 confidence interval shown in brackets).

Species	Family	LM_{xsa} [95% CI]	LM_{xsa} [95% CI]	LM_{xsa} [95% CI]	LM_{xsa} [95% CI]	n
Alstonia muelleriana	Apocynaceae	63.2 [42.3; 94.5]	4361 [2836; 6705]	35.5 [19.0; 66.4]	28.9 [15.7; 53.2]	3
Alstonia scholaris	Apocynaceae	60.8 [49.5; 74.7]	7107 [5548; 9103]	28.4 [17.4; 46.3]	22.8 [13.9; 37.6]	3
Agathis robusta	Araucariaceae	42.1 [30.6; 57.9]	4163 [2790; 6212]	74.3 [58.8; 94.0]	55.5 [43.9; 70.3]	6
Daphnandra repandula	Atherospermataceae	28.2 [22.3; 35.6]	1519 [1180; 1955]	20.4 [15.9; 26.3]	9.6 [7.5; 12.4]	7
Gillbeea adenopetala	Cunoniaceae	24.7 [4.9; 124.7]	2078 [416; 10374]	1.9 [0.5; 6.8]	1.5 [0.4; 5.6]	3
Pseudoweinmannia lachnocarpa	Cunoniaceae	53.8 [45.1; 64.2]	3599 [3230; 4009]	24.4 [21.3; 27.8]	17.6 [15.6; 19.8]	3
Pullea stutzeri	Cunoniaceae	75.5 [62.5; 91.3]	5849 [4800; 7128]	30.2 [23.7; 38.5]	19.5 [14.4; 26.3]	7
Aleurites rockinghamensis	Euphorbiaceae	39.9 [30.7; 51.9]	3762 [2821; 5016]	58.9 [35.8; 96.7]	45.5 [23.5; 88.0]	7
Cleistanthus myrianthus	Euphorbiaceae	25.6 [18.9; 34.6]	2383 [1751; 3244]	12.4 [8.3; 18.6]	8.8 [6.2; 12.6]	3
Cleistanthus semiopacus	Euphorbiaceae	62.4 [42.8; 91.0]	2487 [1465; 4223]	25.3 [20.4; 31.4]	14.7 [11.9; 18.1]	3
Croton insularis	Euphorbiaceae	44.1 [18.6; 104.6]	4123 [1687; 10079]	34.8 [13.7; 88.3]	28.7 [11.3; 72.8]	3
Rockinghamia angustifolia	Euphorbiaceae	79.6 [63.9; 99.1]	5535 [4311; 7106]	17.2 [14.6; 20.2]	13.1 [11.3; 15.2]	3
Acacia celsa	Fabaceae	52.5 [45.3; 60.8]	4543 [3898; 5296]	28.6 [23.3; 35.1]	18.7 [15.2; 23.0]	6
Castanospermum australe	Fabaceae	41.9 [24.6; 71.5]	3005 [1802; 5010]	3.8 [1.1; 13.3]	2.7 [0.8; 9.7]	3
Homalium circumpinnatum	Flacourtiaceae	56.5 [46.8; 68.1]	6519 [5356; 7934]	40.4 [27.9; 58.6]	31.4 [21.6; 45.6]	4
Cinnamomum laubatii	Lauraceae	54.3 [37.4; 78.8]	6912 [4588; 10412]	41.3 [31.1; 54.8]	32.7 [23.8; 45.0]	3
Cryptocarya mackinnoniana	Lauraceae	33.9 [26.5; 43.5]	3676 [2868; 4712]	56.4 [47.6; 66.8]	41.7 [35.3; 49.3]	6
Cryptocarya murrayi	Lauraceae	40.5 [33.1; 49.5]	4148 [3381; 5089]	69.8 [54.4; 89.6]	51.2 [39.9; 65.6]	6
Endiandra leptodendron	Lauraceae	91.0 [48.3; 171.3]	5111 [2514; 10393]	35.3 [22.1; 56.3]	20.2 [9.9; 41.1]	4
Endiandra monothyra subsp. *monothyra*	Lauraceae	45.6 [20.8; 99.9]	2949 [1337; 6506]	18.5 [12.5; 27.5]	14.8 [10.3; 21.3]	3
Litsea leefeana	Lauraceae	37.6 [24.5; 57.8]	6178 [4081; 9353]	34.4 [22.4; 52.6]	28.8 [18.7; 44.2]	6
Neolitsea dealbata	Lauraceae	81.6 [64.7; 102.9]	6024 [4782; 7588]	35.1 [25.6; 48.2]	26.2 [18.9; 36.3]	6
Argyrodendron peralatum	Malvaceae	14.1 [11.0; 18.1]	2791 [2181; 3572]	16.6 [10.7; 25.7]	12.8 [8.4; 19.5]	7
Franciscodendron laurifolium	Malvaceae	52.5 [43.0; 64.1]	4042 [3037; 5379]	58.5 [49.3; 69.3]	44.6 [37.5; 53.0]	6
Steganthera laxiflora	Monimiaceae	34.9 [23.2; 52.5]	3313 [2140; 5130]	30.3 [20.4; 45.1]	24.7 [16.6; 36.8]	3
Myristica globosa subsp. *globosa*	Myristicaceae	64.4 [44.6; 93.2]	6444 [4450; 9332]	61.6 [47.4; 80.1]	43.2 [31.6; 59.1]	6
Gossia hillii	Myrtaceae	65.2 [56.5; 75.2]	4242 [3679; 4890]	31.5 [25.5; 38.9]	21.9 [17.5; 27.4]	6

Species	Family	LM_{xsa} [95% CI]	LM_{xsa} [95% CI]	LM_{xsa} [95% CI]	LM_{xsa} [95% CI]	n
Syzygium sayeri	Myrtaceae	59.2 [50.1; 70.0]	4277 [3542; 5164]	20.5 [17.2; 24.5]	12.1 [9.9; 14.8]	7
Cardwellia sublimis	Proteaceae	30.2 [21.1; 43.3]	2947 [1947; 4462]	32.0 [22.7; 45.2]	22.5 [15.9; 31.8]	5
Darlingia darlingiana	Proteaceae	37.6 [30.5; 46.2]	3592 [2961; 4356]	31.3 [23.6; 41.4]	21.1 [15.9; 27.9]	5
Alphitonia petriei	Rhamnaceae	50.5 [23.9; 106.6]	3741 [1634; 8568]	24.2 [17.3; 33.7]	18.9 [13.3; 26.8]	3
Alphitonia whitei	Rhamnaceae	41.6 [33.7; 51.4]	2502 [2059; 3039]	71.1 [48.2; 104.8]	50.1 [33.9; 74.1]	6
Acronychia acidula	Rutaceae	38.2 [33.5; 43.7]	2834 [2425; 3312]	53.0 [45.2; 62.1]	31.5 [26.3; 37.8]	6
Acronychia laevis	Rutaceae	61.0 [30.7; 121.0]	4697 [2133; 10342]	33.4 [26.9; 41.5]	25.3 [19.7; 32.5]	3
Brombya platynema	Rutaceae	39.5 [32.9; 47.4]	3615 [3023; 4324]	28.9 [23.9; 35.1]	20.5 [15.8; 26.8]	8
Dinosperma erythrococcum	Rutaceae	54.9 [39.6; 76.0]	5915 [4048; 8643]	46.5 [34.2; 63.2]	34.5 [25.1; 47.5]	3
Flindersia bourjotiana	Rutaceae	27.9 [19.1; 40.8]	2142 [1482; 3095]	57.6 [45.0; 73.8]	37.5 [29.3; 48.0]	6
Flindersia brayleyana	Rutaceae	52.7 [35.3; 78.8]	5559 [3791; 8151]	39.1 [25.6; 59.5]	31.2 [19.2; 50.7]	6
Flindersia pimenteliana	Rutaceae	61.3 [53.2; 70.5]	5745 [4565; 7229]	33.7 [22.1; 51.5]	27.6 [18.4; 41.4]	3
Castanospora alphandii	Sapindaceae	72.6 [28.5; 185.4]	5592 [2352; 13293]	47.6 [12.2; 185.5]	33.8 [8.8; 130.0]	3
Dendrocnide photinophylla	Urticaceae	46.6 [37.9; 57.4]	7777 [6146; 9842]	35.4 [25.1; 49.8]	28.3 [20.0; 40.0]	6

Table S5 Details for linear regressions between traits and stem diameter growth rates (95th percentile and mean for trees of all sizes, and 95th percentile for trees between 10 and 30cm dbh).

	Growth Rate Measure								
**Regression functions**	**95th percentile (all sizes)**			**Mean (all sizes)**			**95th percentile (size restricted 10-30cm dbh)**		
	**R^2**	**slope**	**p**	**R^2**	**slope**	**p**	**R^2**	**slope**	**p**
Leaf and wood traits									
log (GR) ~ log (SLA)	0.21	-0.75	0.002	0.34	-1.05	<0.001	0.23	-0.78	0.002
log (GR) ~ A_{area}	0.10	0.02	0.050	0.09	0.03	0.055	0.09	0.02	0.058
log (GR) ~ log (P_{area})	0.22	0.60	0.002	0.22	0.67	0.002	0.21	0.59	0.003
log (GR) ~ log (N_{area})	0.19	0.78	0.004	0.17	0.82	0.007	0.15	0.70	0.011
log (GR) ~ Trunk WD	0.17	-0.59	0.007	0.11	-0.52	0.038	0.12	-0.50	0.029
log (GR) ~ Branch WD	0.09	-0.56	0.054	0.04	-0.43	0.199	0.03	-0.32	0.292
Branch biomass allocation metrics (*at a distance of 100cm from branch tip)*									
log (GR) ~ log (LM:WM)	0.15	0.40	0.012	0.08	0.33	0.07	0.10	0.32	0.048
log (GR) ~ log (LM:SM)	0.27	0.55	<0.001	0.18	0.50	0.006	0.18	0.45	0.006
log (GR) ~ log (LA:WM)	0.009	0.10	0.549	0.006	-0.09	0.623	0.0001	0.01	0.953
log (GR) ~ log (LA:SM)	0.05	0.25	0.149	0.002	0.06	0.761	0.01	0.13	0.473
Branch biomass allocation metrics (*at $100mm^2$ cross sectional area)*									
log (GR) ~ log (LM:WM)	0.29	0.39	0.0002	0.21	0.37	0.003	0.19	0.32	0.004
log (GR) ~ log (LM:SM)	0.34	0.41	<0.0001	0.26	0.41	0.001	0.23	0.34	0.002
log (GR) ~ log (LA:WM)	0.14	0.28	0.017	0.05	0.19	0.166	0.07	0.20	0.106
log (GR) ~ log (LA:SM)	0.19	0.34	0.004	0.09	0.26	0.059	0.10	0.25	0.046

Table S6 Matrix of Pearson product-moment correlation coefficients between traits (LM:SM estimated at both a standard distance and a standard cross-sectional area).

^ 0.05 < p < 0.1, * p < 0.05, **p < 0.001, *** p < 0.0001

	log (SLA)	A_{area}	log (P_{area})	log (N_{area})	WD	log (LM:SM dist)
A_{area}	-0.38*					
log (P_{area})	-0.27^	0.42*				
log (N_{area})	-0.35*	0.64***	0.67***			
WD	-0.15	-0.04	-0.17	-0.19		
log (LM:SM dist)	-0.38*	0.26^	0.35*	0.24	-0.17	
log (LM:SM xsa)	-0.43*	0.36*	0.50**	0.41*	-0.29^	0.89***

Table S7 Axis loadings and explained variance of the first three components of a principal component analysis including all traits (LM:SM at a standard cross-sectional area).

Traits	PC1	PC2	PC3
	Axis loadings		
log (SLA)	0.34	-0.51	-0.53
WD	0.15	0.82	-0.18
log (LM:SM)	-0.43	-0.15	0.59
A_{area}	-0.44	0.18	-0.38
log (P_{area})	-0.47	-0.12	-0.20
log (N_{area})	-0.51	-0.03	-0.40
Eigen values	2.84	1.17	0.79
Variance explained	47.3%	19.5%	13.2%

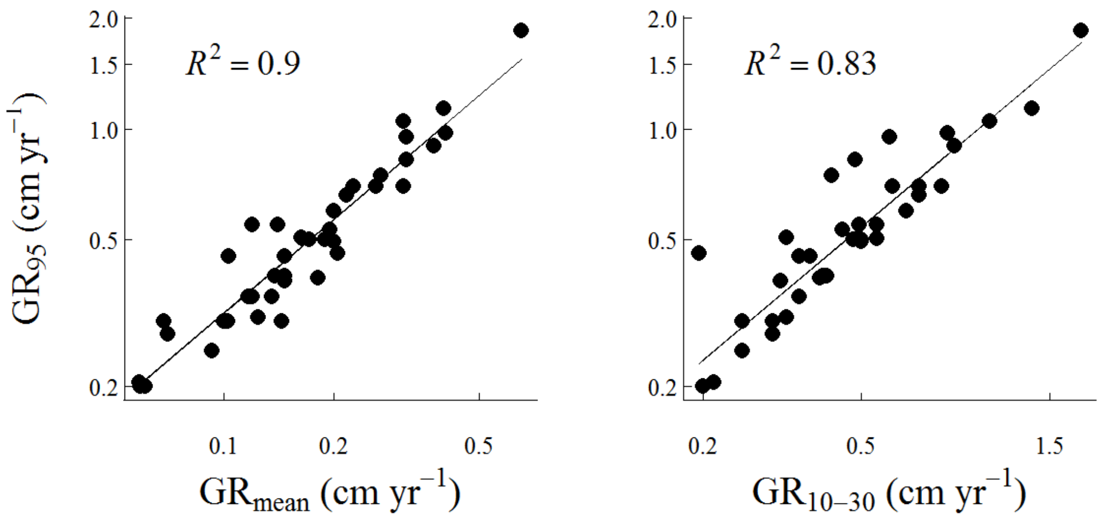

Figure S1 Linear regression relationships between GR_{95} and the two other estimates of growth rate, namely mean growth rate (GR_{mean}) and 95th percentile growth rate of individuals within a restricted sizeclass of 10-30 cm diameter (GR_{10-30}). Relationships are for 41 rainforest species, based on data in Table S1.

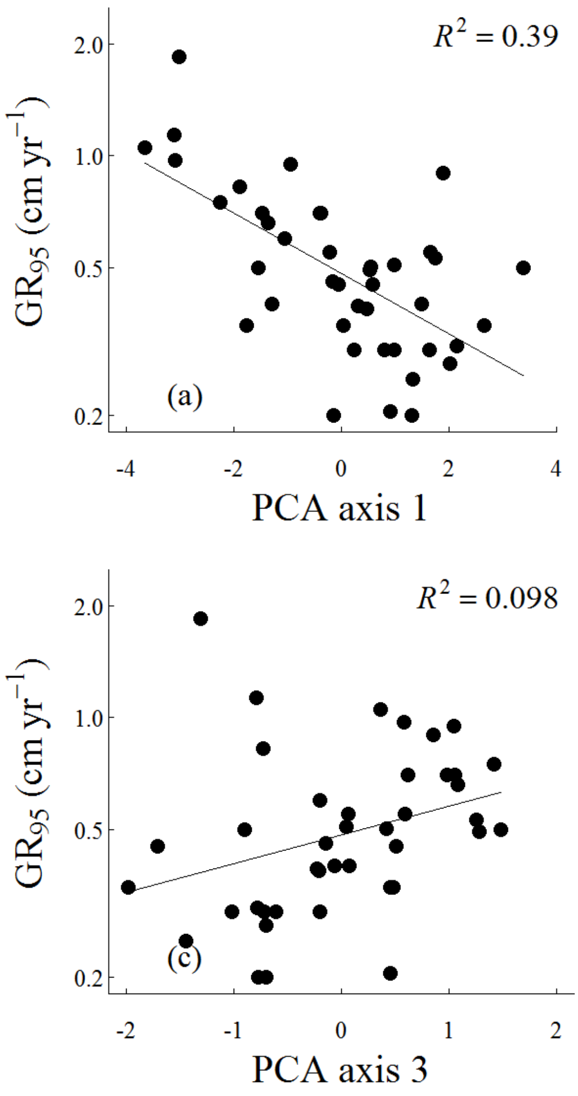

Figure S2 Linear regression relationships between GR_{95} and PCA axes (a) principal components axis 1, b) principal components axis 2, and c) principal components axis 3. Black trend lines indicate significant regression relationships, grey lines show non-significant relationships.

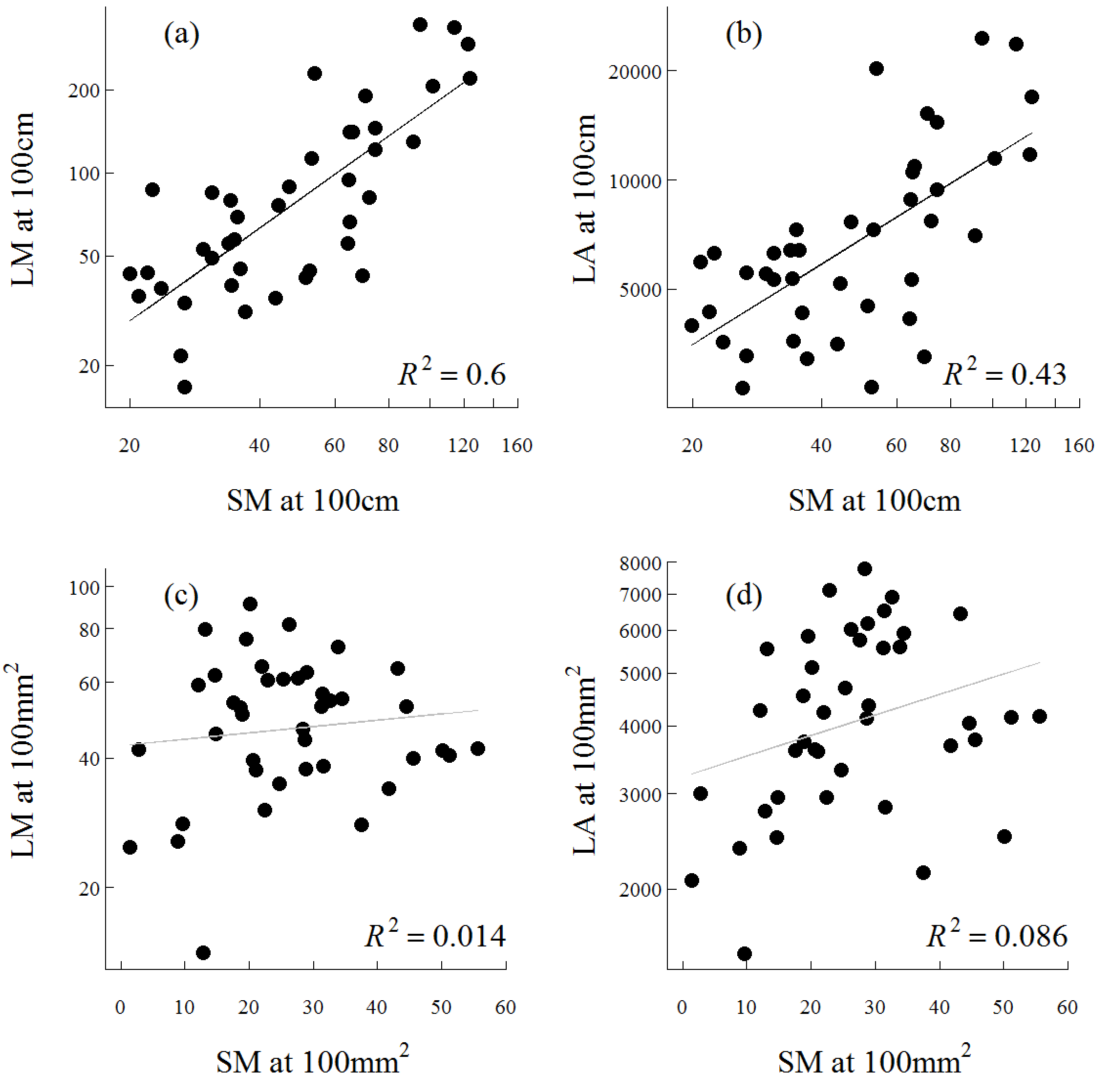

Figure S3 Linear regression relationships between leaf and sapwood components (leaf mass (LM), leaf area (LA) and sapwood mass (SM)) at a standardised distance from tip (a, b) and a standardised cross-sectional area (c, d). All variables except for sapwood mass at a standardised cross-sectional area are log transformed. Black trend lines indicate significant regression relationships, grey lines show non-significant relationships.
